# Nutrient Removal and Oxidative Response of Barley to Eutrophic Stress

**DOI:** 10.3390/plants14233595

**Published:** 2025-11-25

**Authors:** Jing Sun, Minghui Hou, Yanliang Sun, Shahbaz Khan, Kaixin Yang, Qianbing Zhang

**Affiliations:** College of Animal Science & Technology, Shihezi University, Shihezi 832000, China

**Keywords:** hydroponics, barley, growth performance, nutritional quality, nitrogen and phosphorus removal rate

## Abstract

Eutrophication caused by excessive nitrogen (N) and phosphorus (P) inputs threatens aquatic ecosystems and requires sustainable solutions. Barley (*Hordeum vulgare* L.) is a fast-growing forage crop with potential for removing nutrients in eutrophic waters; however, a comprehensive understanding of its physiological responses across a wide gradient of concurrent N and P levels is still developing. To investigate this, a 7-day hydroponic experiment was conducted: six N + P enrichment levels, control (0), 70 + 7, 140 + 14, 280 + 28, 560 + 56, and 1120 + 112 mg·L^–1^ of total N and P (TN:TP fixed at 10:1), were prepared in hydroponic culture and their effect on seed germination, growth, nutritional quality, photosynthetic pigments, antioxidant activity, and nutrient removal efficiency were studied. Results showed that early germination was inhibited under nutrients and enriched conditions, but this recovered by day 3. A moderate nutrient supply (NP 70 + 7) promoted seedling growth, resulting in maximum plant height, fresh weight, crude protein (6.6%), ether extract (6.1%), and chlorophyll a (10.9%) compared to the control. Root growth was best in control, while high nutrient stress (NP 1120 + 112) led to the highest mortality (23.5%). This mortality was linked to a severe suppression of the enzymatic antioxidant system (SOD, POD, and CAT), indicating a collapse of primary oxidative defense under extreme stress. Nutritional quality improved under NP 140 + 14, which yielded the lowest NDF and ADF and the highest chlorophyll b (15%) and glutathione content (14.9%). Antioxidant enzymes (SOD, POD, and CAT) were most active in the control and declined with increasing N + P levels, while glutathione peaked under NP 140 + 14. This indicates a potential shift in the plant’s defense strategy, where glutathione plays a key role in mediating tolerance to moderate eutrophic stress. Nutrient removal analysis showed that N removal efficiency was highest under NP 70 + 7 (53.4%), whereas P removal decreased consistently with rising concentrations. Overall, barley grass seedlings tolerated N + P levels up to NP 280 + 28 while maintaining growth and nutrient uptake, a resilience facilitated by its dynamic antioxidant response, suggesting their potential role in phytoremediation of eutrophic waters.

## 1. Introduction

Water is the foundation of agricultural production and ecological stability. Yet, in many parts of the world, especially in inland arid and semi-arid regions, water scarcity has become a pressing challenge. The Xinjiang Uygur Autonomous Region, situated in northwest China, is a typical inland arid area characterized by extremely limited water availability [1,2]. Annual precipitation in Xinjiang is only about one-twelfth of the national average, and its distribution is highly uneven [3]. In addition, high summer temperatures, frequent strong winds, and intense evaporation, combined with the limited recharge capacity of groundwater, exacerbate the imbalance between water supply and demand [4].

To compensate for the harsh climatic conditions and low soil fertility, many farmers in arid regions have increasingly relied on chemical fertilizers to boost crop yields. Statistics indicate that fertilizer application rates in Xinjiang have already exceeded the national average [5]. While this practice has temporarily improved yields, excessive fertilizer use has introduced severe environmental problems, particularly water pollution [5]. Nutrient runoff from agricultural fields has accelerated the accumulation of nitrogen (N) and phosphorus (P) in rivers, lakes, and reservoirs, creating favorable conditions for algal blooms and eutrophication [6]. Once eutrophication occurs, it disrupts the balance of aquatic ecosystems, reduces water clarity, depletes dissolved oxygen, and can ultimately lead to mass fish mortality and biodiversity loss [7].

Eutrophication of freshwater ecosystems is now recognized as a global environmental challenge [8]. Its root cause is nutrient over enrichment, mainly from domestic wastewater, industrial discharge, and excessive fertilizer use in agriculture. Modern agricultural intensification has significantly increased nitrogen (N) and phosphorus (P) inputs into aquatic systems, making agriculture a dominant driver of eutrophication worldwide [9]. Once established, eutrophication is difficult to reverse and often requires costly remediation measures. Therefore, identifying efficient, low-cost, and sustainable biological solutions to remove excess nutrients from water has become an urgent research priority [10].

In recent decades, aquatic macrophytes and some terrestrial plants have been tested as phytoremediation tools for nutrient removal [11]. Species such as water hyacinth, water lily, and ramie have demonstrated potential to absorb N and P from contaminated water [12,13,14,15]. However, these plants face several limitations. Their ecological adaptability is narrow, their growth is often seasonal, and they provide limited economic benefits, making them less suitable for widespread or long-term application [16,17]. To achieve both ecological and economic benefits, it is necessary to identify stress-tolerant species, produce a large shoot surface area [16], be widely adaptable, and have valuable byproducts, such as animal feed (Phang et al., 2024) [18].

In this context, grasses have attracted attention. Members of the Poaceae family, such as rye and barley grass, are not only widely cultivated but also exhibit high stress tolerance and potential to remediate polluted water [19,20,21,22]. After use in water treatment, they can serve as valuable forage resources, thus coupling ecological restoration with agricultural production. This dual-purpose advantage enhances their feasibility for large-scale adoption in regions struggling with both nutrient pollution and feed shortages [21].

Barley (*Hordeum vulgare* L.) is particularly promising. Barley grass, harvested at a height of 15–20 cm before the heading stage, is highly digestible, rich in protein, and contains high fiber, minerals, beneficial phytochemicals, and considerable energy, making it a nutritious feed resource for livestock [23]. Moreover, hydroponic barley cultivation has been increasingly promoted as a rapid and space-efficient technique to produce high-quality forage. In hydroponic systems, barley grass roots are directly immersed in nutrient-rich solutions, allowing for rapid nutrient absorption and biomass accumulation [24,25]. Importantly, during hydroponic growth, barley not only absorbs nutrients directly from the water but also releases root exudates that may inhibit algal proliferation, thereby contributing to improved water quality. With its short growth cycle, high productivity, and small land footprint, hydroponic barley grass offers unique advantages as an ecological restoration material with significant economic returns [26,27].

Despite these promising characteristics, current research on the use of grass for ecological restoration has largely focused on ryegrass [28,29,30], while studies on barley grass seedlings remain scarce. The capacity of barley grass to simultaneously grow under eutrophic conditions, improve water quality by removing pollutants, and provide high-value forage has not been systematically investigated. Furthermore, only a few studies have explored the physiological mechanisms underlying the response of grasses to high nutrient stress, including antioxidant defense systems, photosynthetic pigment dynamics, and nutrient uptake [28].

Therefore, there is a clear need to comprehensively evaluate the efficiency of barley grass seedlings in removing nitrogen and phosphorus from water, especially in regions like Xinjiang, where water scarcity, fertilizer overuse, and eutrophication converge. The novelty of this work lies in linking physiological mechanisms (pigment dynamics, antioxidant responses) with practical outcomes (biomass quality and nutrient removal), thereby identifying the optimal N–P ranges for sustainable application in nutrient-rich waters.

To address these questions, a hydroponic experiment was designed with six N–P treatments (0, NP 70 + 7, NP 140 + 14, NP 280 + 28, NP 560 + 56, NP 1120 + 112 mg·L^−1^), representing a gradient of nutrient enrichment. Seed germination, growth performance, nutritional quality, pigment accumulation, antioxidant capacity, and nutrient removal efficiency were analyzed after seven days of cultivation. By integrating agronomic, physiological, and ecological indicators, this research provides new insights into the feasibility of hydroponic barley grass seedlings as a low-cost, dual-purpose tool for mitigating eutrophication while producing valuable feed. This work addresses an urgent environmental and agricultural challenge by evaluating barley seedlings as a model system to balance water quality improvement and forage production.

## 2. Materials and Methods

### 2.1. Plant Material

Barley (*Hordeum vulgare* L.) Ganpi No. 4 was used in this study. Seeds were provided by the Agricultural College of Shihezi University, Xinjiang, China. This is a high yielding variety with strong resistance to abiotic stresses, making it suitable for controlled experiments under nutrient enriched water conditions.

### 2.2. Experimental Site and Growth Conditions

The experiment was conducted in hydroponic trays from June to August 2023 at the College of Animal Science and Technology, Shihezi University, Xinjiang, China. An RXZ-500D intelligent artificial climate chamber manufactured (RXZ-500D, Ningbo Jiangnan Instrument Factory, Ningbo, Zhejiang, China) was used to maintain controlled environmental conditions. The chamber was set at a 12 h light/12 h dark photoperiod, with temperatures of 25 ± 1 °C (day) and 15 ± 1 °C (night), 50–70% relative humidity, and a PAR of 250 µmol·m^−2^·s^−1^.

### 2.3. Eutrophic Water Preparation and Treatments

Nutrient solutions simulating eutrophic water were prepared according to the Surface Water Environmental Quality Standard (GB 3838-2002 [31]), which defines the typical ratio of total nitrogen (N) to total phosphorus (P) in surface water as ranging between 5:1 and 10:1 [30]. In this study, the TN:TP ratio was fixed at 10:1.

Urea [CO(NH_2_)_2_] and calcium superphosphate [Ca(H_2_PO_4_)_2_·H_2_O] were dissolved in distilled water to make five NP enrichment levels fixed at a 10:1 ratio. Urea was selected as the primary nitrogen source to better simulate agricultural runoff, where it is the predominant N fertilizer, and to assess plant tolerance to the associated ammonium stress that can arise from its hydrolysis. Nitrogen was expressed on an elemental basis as mg N·L^−1^ (Kjeldahl-N), while phosphorus was reported as mg P L^−1^. Treatments were as follow: (i) NP 70 + 7 (70 mg·L^−1^ N + 7 mg·L^−1^ P), (ii) NP 140 + 14 (140 mg·L^−1^ N + 14 mg·L^−1^ P), (iii) NP 280 + 28 (280 mg·L^−1^ N + 28 mg·L^−1^ P), (iv) NP 560 + 56 (560 mg·L^−1^ N + 56 mg·L^−1^ P), and (v) NP 1120 + 112 (1120 mg·L^−1^ N + 112 mg·L^−1^ P), distilled water served as the control treatment. The pH of all solutions was adjusted to 7.0 using dilute NaOH or HCl before use. Each treatment was replicated three times.

### 2.4. Seed Sterilization and Germination Assay

Seeds were surface sterilized by immersion in a 1% (*w*/*v*) limewater solution for 6 h, followed by thorough rinsing with distilled water. For each treatment, 200 seeds were evenly placed on two layers of sterilized filter paper in 9 cm diameter Petri dishes. Each dish received 10 mL of the respective treatment solution. Petri dishes were incubated in the climate chamber under the conditions described in Section 2.2.

### 2.5. Seedling Growth Assay

Seed germination (%) was monitored on day 1 and day 3 after sowing. A seed was considered germinated once the radicle length exceeded 2 mm. At 72 h post imbibition, uniform seedlings were transferred to hydroponic trays (32 × 24 × 4.5 cm). Each tray contained 1000 seedlings and 1.5 L of the designated treatment solution. Solutions were replaced every 48 h. After 4 days (i.e., day 7 from sowing), seedlings were gently rinsed and blotted dry with absorbent paper.

A seedling was considered dead if more than two thirds of its shoot was wilted. The mortality rate (%) was calculated per tray. From each tray, 20 seedlings were randomly selected for measurement of growth parameters. Root length and shoot length were measured using a digital caliper (Model 500-196-20, Mitutoyo Corp., Kawasaki, Japan), and fresh weight was determined with an electronic balance (Model ME204/02, Mettler Toledo, Greifensee, Switzerland).

For dry biomass analysis, 100 g of fresh barley seedling material from each treatment was blanched at 105 °C for 30 min, then oven dried at 65 °C to a constant weight. The dried samples were ground into a fine powder using a mill and then passed through an 80-mesh sieve for storage and subsequent analysis.

### 2.6. Photosynthetic Pigment Determination

Chlorophyll a, chlorophyll b, and carotenoids were determined by the ethanol extraction method [32]. Absorbance at 470, 649, and 665 nm was recorded with a UV visible spectrophotometer (Model UV-2600, Shimadzu Corporation, Kyoto, Japan), and pigment concentrations were calculated using standard equations.

### 2.7. Antioxidant Enzyme Activity and Glutathione Content

Crude enzyme extracts were prepared on ice from fresh seedling tissue (1:10, *w*/*v*) using the extraction buffer supplied with the kit. The superoxide dismutase (SOD), peroxidase (POD), and catalase (CAT) activities and glutathione (GSH) content were quantified using commercial assay kits (Nanjing Jiancheng Bioengineering Institute, Nanjing, China), following the manufacturer’s manuals. The general principles of these assays follow [33] for SOD, [34] for POD, and [35] for CAT, which are widely adopted in plant physiology studies.

### 2.8. Nitrogen and Phosphorus Removal from Solution

A separate experiment was conducted to determine the removal efficiency of total nitrogen (TN) and total phosphorus (TP) from the culture medium by barley seedlings. Approximately 300 g of sterilized seeds were soaked in distilled water overnight and then evenly distributed in hydroponic trays. Once the roots reached 1 cm in length, 1.5 L of nutrient solution (prepared as described in Section 2.3) was added to the trays.

After 4 days of cultivation, the residual TN and TP concentrations in the solutions were measured. TN was determined using the Kjeldahl method [36], while TP was quantified by the ammonium molybdate spectrophotometric method [37]. The nutrient removal rate (%) was calculated as:

Removal rate (%) = (C_0_ − C_t_)/C_0_ × 100

where C_0_ and C_t_ are initial and final concentrations (mg·L^−1^) after 4 days.

### 2.9. Proximate Composition of Seedlings (DM—Basis Nutrients)

#### 2.9.1. Sample Preparation and Dry Matter (DM)

Bulk seedling samples from each tray were quickly chopped, mixed, and subsampled. Dry matter (DM, % of fresh weight, FW) was determined by drying fresh subsamples at 105 °C for 30 min, followed by 65 °C to a constant mass (AOAC 2016 [38]; GB/T 6435 [39]).

DM (% of FW) = (Dry weight/Fresh weight) × 100

Dried samples were ground to pass through an 80-mesh sieve and stored in airtight bags for subsequent chemical analyses.

#### 2.9.2. Neutral and Acid Detergent Fiber (NDF, ADF)

NDF and ADF were measured by the Van Soest detergent fiber method using a ANKOM A200 fiber analyzer (ANKOM A200, ANKOM Technology, Macedon, NY, USA) (e.g., ANKOM; reagents per Van Soest) (Van Soest et al., 1991 [40]). Results are expressed as % of DM.

#### 2.9.3. Crude Protein (CP)

Total nitrogen was determined by the Kjeldahl digestion method. Dried, ground barley seedling samples (0.5 g) were digested with concentrated H_2_SO_4_ in the presence of a catalyst (e.g., H_2_SO_4_ + CuSO_4_). The digested solution was distilled with excess NaOH, and the released NH_3_ (trapped in boric acid solution) was titrated with standard H_2_SO_4_ or HCl. Crude protein (CP) was calculated as N × 6.25 (AOAC 2016 [38]; GB/T 6432 [41]).

#### 2.9.4. Ether Extract (EE)

Ether extract was determined gravimetrically by continuous extraction with petroleum ether in a Soxhlet apparatus (Model SXT-02, Shanghai Hongji Instrument Co., Ltd., Shanghai, China) (AOAC 2016 [38]; GB/T 6433 [42]). Approximately 2 g of dried, ground barley seedling sample was extracted with petroleum ether (boiling point: 40–60 °C) in a Soxhlet extractor for 6—8 h. The ether was evaporated, and the residue (fat fraction) was dried to constant weight at 105 °C.

Ether extract content was calculated using a formula and expressed as % of dry matter (DM):

Ether extract (% of DM) = (W_1_ − W_0_)/Ws × 100

where W_1_= weight of flask + fat residue (g), W_0_= weight of empty flask (g), and Ws = weight of sample (g, dry basis).

#### 2.9.5. Soluble Sugars

About 0.5 g of dried barley seedling powder was extracted with 80% ethyl acetate. The extract was centrifuged, and the supernatant was collected. Aliquots of the extract were mixed with anthrone sulfuric acid reagent and heated in a water bath (95 °C, 10 min). After cooling, absorbance was measured at 620 nm using a spectrophotometer. The soluble sugar concentration was calculated using a glucose standard curve.

### 2.10. Experimental Design and Statistics

Treatments were arranged in a completely randomized design with three biological replicates (one Petri plate or one tray = one replicate). Data were checked for normality and variance homogeneity; one way ANOVA was performed, followed by Tukey’s HSD (α = 0.05). Different letters above bars/points indicate significant differences among treatments. Results are presented as mean ± SE (n = 3). Statistical analyses were performed using R (version 4. x) or SPSS (version 26). Pearson correlation analysis was conducted using all data points from the different treatments. The correlation coefficients were calculated, and a correlation matrix heatmap was generated to visualize the strength and direction of the linear relationships. The significance of the correlations was assessed at the *p* < 0.05 level.

## 3. Results

### 3.1. Effects of Different Nitrogen and Phosphorus Levels on Barley Seed Germination Rate

Different nitrogen and phosphorus enrichments markedly suppressed the first day germination rate of barley seeds, and the degree of inhibition increased with nutrient concentration (Table 1). Compared with the control, germination declined by 24%, 25.7%, 42.7%, 44%, and 63.5% on day 1 under NP 70 + 7, NP 140 + 14, NP 280 + 28, NP 560 + 56, and NP 1120 + 112, respectively. The most severe inhibition occurred under NP 1120 + 112, where nearly two thirds of the plants experienced a reduction in their germination rate.

In contrast, the third day germination rate did not differ significantly among treatments (*p* > 0.05). This indicates that while high nitrogen and phosphorus concentrations delayed early germination, they did not ultimately prevent seed germination once sufficient time was allowed. However, different nitrogen and phosphorus levels had no significant effect on the germination rate on the third day (*p* > 0.05).

### 3.2. Effects of Nitrogen and Phosphorus Levels on Growth Performance and Mortality of Barley Seedlings

**Figure 1:**
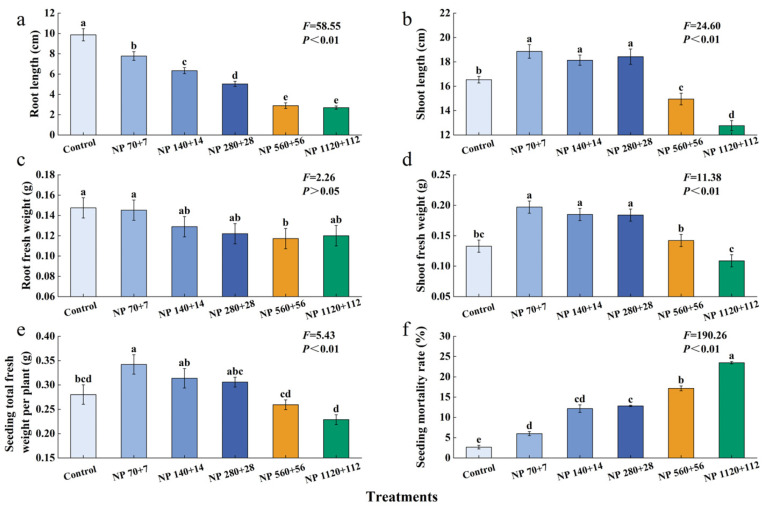
Effects of different nitrogen and phosphorus levels on the production performance and mortality rate of barley grass. Treatments: Control (distilled water); NP 70 + 7 = 70 mg·L^−1^ N + 7 mg·L^−1^ P; NP 140 + 14 = 140 mg·L^−1^ N + 14 mg·L^−1^ P; NP 280 + 28 = 280 mg·L^−1^ N + 28 mg·L^−1^ P; NP 560 + 56 = 560 mg·L^−1^ N + 56 mg·L^−1^ P; NP 1120 + 112 = 1120 mg·L^−1^ N + 112 mg·L^−1^. *p* Values represent mean ± SE (n = 3). Different letters indicate significant differences among treatments according to Tukey’s HSD test (*p* < 0.05). (**a**) Root length; (**b**) Shoot length; (**c**) Root fresh weiht; (**d**) Shoot fresh weihjt; (**e**) Seeding total fresh weight per plant; (**f**) Seeding mortality rate.

Moderate nitrogen and phosphorus levels significantly enhanced the growth of barley seedlings (Figure 1). Plant height, shoot fresh weight, and aboveground biomass exhibited a unimodal trend, increasing at lower concentrations and declining at higher concentrations (Figure 1b,d,e). The highest values for these traits were observed under NP 70 + 7, and the lowest at NP 1120 + 112.

In contrast, root traits showed a consistent inhibitory response under increasing nitrogen and phosphorus concentrations. Root length showed the most drastic reduction, and all NP levels caused significant reductions (Figure 1a). A significant reduction in root weight was only observed at NP 560 + 56 (Figure 1c).

All levels of NP caused a significant increase in seedling mortality (Figure 1f). The lowest seed mortality rate (2.7%) was observed at the control. In contrast, NP rates of 70 + 7, 140 + 14, 280 + 28, 560 + 56, and 1120 + 112 caused a 6.0%, 12.2%, 12.8%, 17.2%, and 23.5% increase in mortality rate, respectively, compared to the control.

### 3.3. Effects of Different Nitrogen and Phosphorus Levels on the Nutritional Quality of Barley Grass

**Figure 2:**
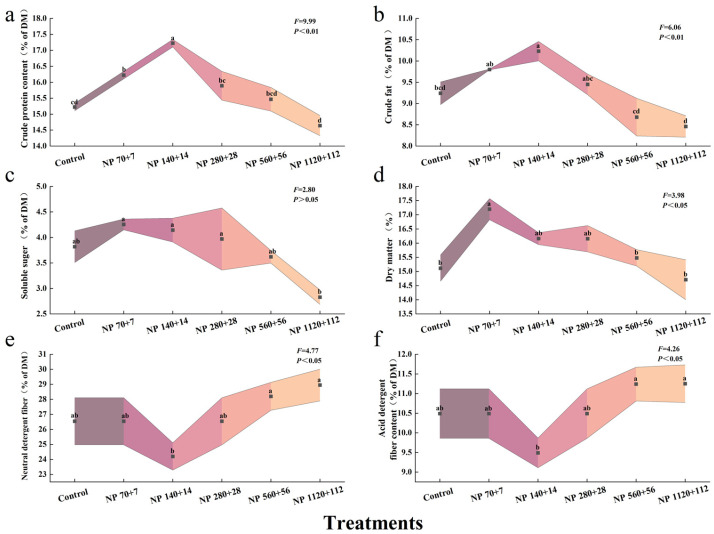
Effects of different nitrogen and phosphorus levels on the quality traits of barley seedlings: (**a**) crude protein content, (**b**) crude fat, (**c**) soluble sugar, (**d**) dry matter, (**e**) neutral detergent fiber, and (**f**) acid detergent fiber. Treatments: Control (distilled water); NP 70 + 7 = 70 mg·L^−1^ N + 7 mg·L^−1^ P; NP 140 + 14 = 140 mg·L^−1^ N + 14 mg·L^−1^ P; NP 280 + 28 = 280 mg·L^−1^ N + 28 mg·L^−1^ P; NP 560 + 56 = 560 mg·L^−1^ N + 56 mg·L^−1^ P; NP 1120 + 112 = 1120 mg·L^−1^ N + 112 mg·L^−1^. *p* Values represent mean ± SE (n = 3). Different letters indicate significant differences among treatments according to Tukey’s HSD test (*p* < 0.05).

Nitrogen and phosphorus enrichment markedly influenced the nutritional composition of barley seedlings (Figure 2). The contents of crude protein, ether extract, soluble sugar, and dry matter all exhibited a unimodal response, increasing at lower nutrient concentrations and declining under higher levels. Compared with the control, crude protein content increased by 6.6% in NP 70 + 7, reaching a maximum increase of 13% in NP 140 + 14, and then declined to −3.8% in NO 1120 + 112 (Figure 2a).

Ether extract content followed a similar pattern, increasing by 6.1% and 10.7% under NP 70 + 7 and NP 140 + 14, respectively, but decreasing by 6.1% and 8.4% at NP 560 + 56 and NP 1120 + 112, respectively (Figure 2b). Soluble sugars were most enhanced under NP 70 + 7 (11.4%) but dropped sharply to −25.6% at NP 1120 + 112 (Figure 2c). Dry matter content also peaked at NP 70 + 7 (13.7%) before decreasing slightly at higher concentrations (Figure 2d).

In contrast, neutral detergent fiber (NDF) and acid detergent fiber (ADF) displayed an opposite trend: both initially decreased and then rose again with increasing nutrient supply (Figure 2e,f). The lowest values for NDF and ADF were recorded under NP 140 + 14, where they were reduced by 8.8% and 9.5%, respectively, compared with the control (*p* < 0.05).

### 3.4. Effects of Nitrogen and Phosphorus Levels on Photosynthetic Pigment Content in Barley Seedlings

**Figure 3:**
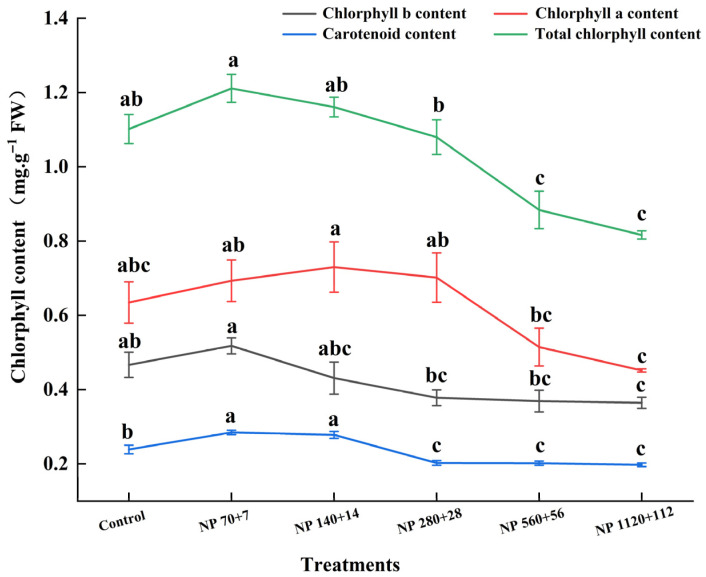
Effects of different nitrogen and phosphorus levels on the photosynthetic pigments of barley seedlings. Treatments: Control (distilled water); NP 70 + 7 = 70 mg·L^−1^ N + 7 mg·L^−1^ P; NP 140 + 14 = 140 mg·L^−1^ N + 14 mg·L^−1^ P; NP 280 + 28 = 280 mg·L^−1^ N + 28 mg·L^−1^ P; NP 560 + 56 = 560 mg·L^−1^ N + 56 mg·L^−1^ P; NP 1120 + 112 = 1120 mg·L^−1^ N + 112 mg·L^−1^. *p* Values represent mean ± SE (n = 3). Different letters indicate significant differences among treatments according to Tukey’s HSD test (*p* < 0.05).

Nitrogen and phosphorus supply significantly affected the accumulation of photosynthetic pigments in barley leaves (Figure 3). Both chlorophyll a and b followed a unimodal trend, increasing at lower nutrient levels and declining sharply at high concentrations. Compared with the control, chlorophyll a increased by 10.6% under NP 70 + 7 but declined by −8.5% to −23.4% across higher treatments. Similarly, chlorophyll b increased by 9.5% under NP 70 + 7 and reached a maximum of 15.9% under NP 140 + 14 and then declined to −28.6% under NP 1120 + 112.

Carotenoid content was most enhanced under NP 70 + 7 (16.7% above control), remained significantly similar to NP 140 + 14, and started declining at higher nutrient levels (*p* < 0.05). Overall, these results suggest that moderate nitrogen and phosphorus supply enhances pigment biosynthesis, whereas excessive levels suppress chlorophyll accumulation while maintaining relatively stable carotenoid levels.

### 3.5. Effects of Different Nitrogen and Phosphorus Levels on the Antioxidant Capacity of Barley Seedlings

**Figure 4:**
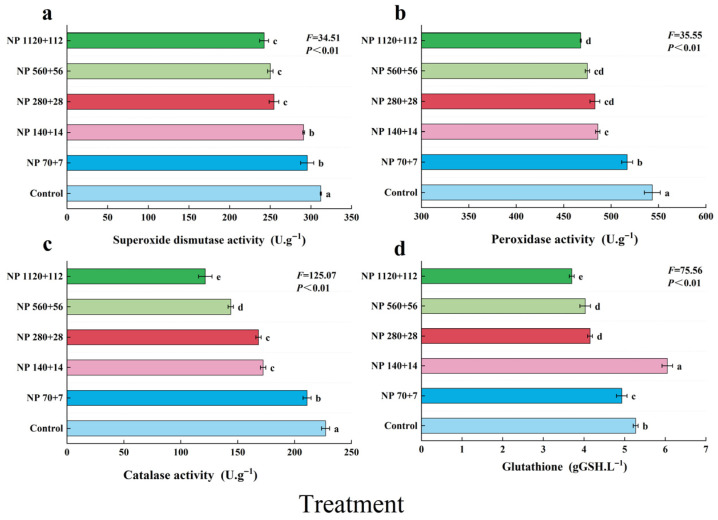
Effects of different nitrogen and phosphorus levels on the antioxidant capacity of barley seedlings. (**a**) superoxide dismutase, (**b**) peroxidase activity, (**c**) catalsae activity, and (**d**) glutathione content. Treatments: Control (distilled water); NP 70 + 7 = 70 mg·L^−1^ N + 7 mg·L^−1^ P; NP 140 + 14 = 140 mg·L^−1^ N + 14 mg·L^−1^ P; NP 280 + 28 = 280 mg·L^−1^ N + 28 mg·L^−1^ P; NP 560 + 56 = 560 mg·L^−1^ N + 56 mg·L^−1^ P; NP 1120 + 112 = 1120 mg·L^−1^ N + 112 mg·L^−1^. *p* Values represent mean ± SE (n = 3). Different letters indicate significant differences among treatments according to Tukey’s HSD test (*p* < 0.05).

Nitrogen and phosphorus enrichment significantly influenced antioxidant defense in barley leaves (Figure 4). SOD, POD, and CAT activities all declined progressively with increasing nutrient concentration, with the highest activities observed in the control (312.1, 543.5, and 227.2 U·g^−1^, respectively). Relative to the control, SOD activity decreased by −5.3% to −22.3% (Figure 4a), POD activity by −4.9% to −13.9% (Figure 4b), and CAT activity by −7.2% to −46.5%, with the steepest decline in CAT under NP 1120 + 112 (Figure 4c).

In contrast, glutathione (GSH) content exhibited a nonlinear response, decreasing initially, peaking under NP 140 + 14 at 6.05 (units) (an increase of 14.9% compared with control), and then declining again at higher nutrient levels (Figure 4d). These results indicate that while excessive nitrogen and phosphorus inputs impair enzymatic antioxidant defenses, moderate nutrient supply may transiently stimulate nonenzymatic antioxidant accumulation.

The progressive decline in key antioxidant enzyme activities (SOD, POD, CAT) under high N-P stress (NP 560 + 56 and NP 1120 + 112), coupled with the subsequent drop in GSH content, indicates a potential collapse of the reactive oxygen species (ROS) scavenging system. This impairment in antioxidant defense is mechanistically linked to the observed physiological damages: the sharp decrease in chlorophyll content (Figure 3) suggests photo-oxidative damage, and the significant increase in seedling mortality (Figure 1f) likely results from irreversible oxidative stress to cellular membranes and metabolites. Conversely, the peak of GSH, a crucial redox buffer, under moderate stress (NP 140 + 14) corresponds with the optimal nutritional quality and lower mortality at this treatment, highlighting its key role in conferring tolerance to moderate eutrophic stress.

### 3.6. Removal Rate of Total Nitrogen and Total Phosphorus by Barley Seedlings

**Figure 5:**
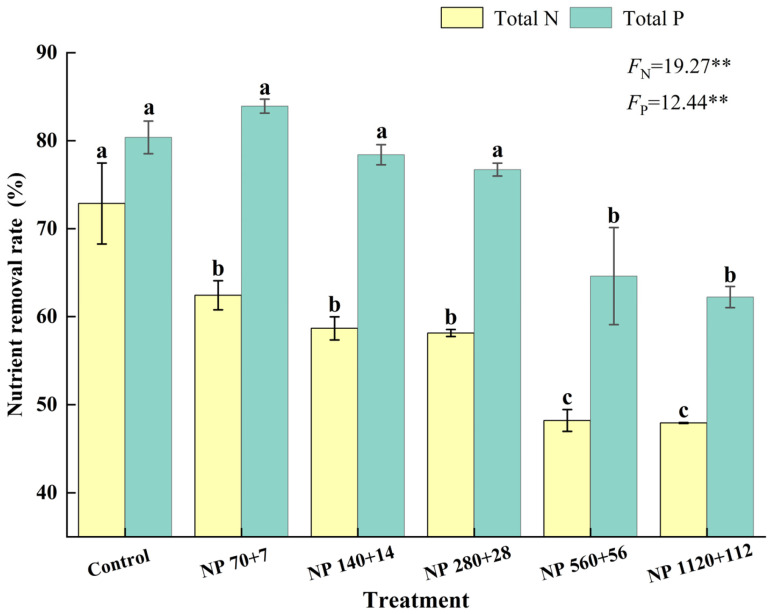
Effects of different nitrogen and phosphorus levels on nitrogen and phosphorus removal rate of barley seedlings. Treatments: Control (distilled water); NP 70 + 7 = 70 mg·L^−1^ N + 7 mg·L^−1^ P; NP 140 + 14 = 140 mg·L^−1^ N + 14 mg·L^−1^ P; NP 280 + 28 = 280 mg·L^−1^ N + 28 mg·L^−1^ P; NP 560 + 56 = 560 mg·L^−1^ N + 56 mg·L^−1^ P; NP 1120 + 112 = 1120 mg·L^−1^ N + 112 mg·L^−1^. *p* Values represent mean ± SE (n = 3). Different letters indicate significant differences among treatments according to Tukey’s HSD test (*p* < 0.05). “**” indicate a highly significant difference (*p* < 0.01).

Nitrogen and phosphorus concentrations significantly influenced the removal of nutrients from water by barley seedlings (Figure 5). The removal rate decreased steadily as nutrient levels increased. The maximum nitrogen removal was observed under NP 70 + 7, where seedlings removed 53.4% of total nitrogen, while NP 140:14 and NP 280:28 were significantly similar to NP 70 + 7.

Similarly, phosphorus removal was highest at NP 70 + 7 and significantly declined at NP 560 + 56 and NP 1120 + 112. Together, these results suggest that barley seedlings are more effective in nitrogen uptake under moderate eutrophic conditions; however, phosphorus uptake efficiency diminishes progressively as external nutrient concentrations increase.

### 3.7. Correlation Between Nutrient Removal, Growth Traits, Nutritional Quality, Pigments, and Antioxidant Capacity

**Figure 6:**
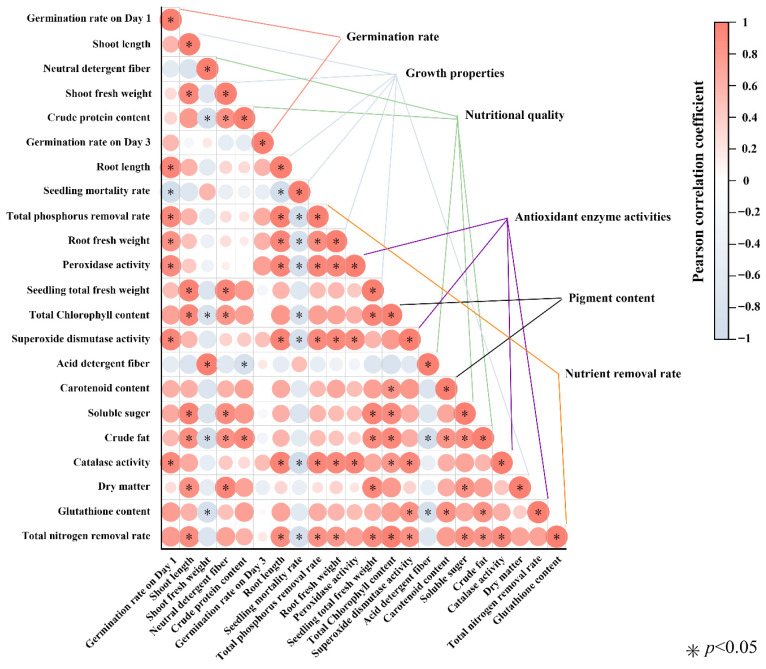
Pearson correlation analysis among germination, growth performance, nutritional quality, photosynthetic pigments, antioxidant enzyme activities, and nutrient removal efficiency in barley seedlings under different nitrogen and phosphorus concentrations. The heatmap shows Pearson correlation coefficients (ranging from –1 to +1, color scale on the right) between measured traits. Red circles indicate positive correlations, and blue circles indicate negative correlations, with circle size proportional to the correlation strength. Asterisks (*) denote statistically significant correlations at *p* < 0.05.

To further explore the relationships among growth performance, nutritional quality, photosynthetic pigments, antioxidant defense, and nutrient removal efficiency under different nitrogen and phosphorus treatments, a Pearson correlation analysis was performed (Figure 6).

The total nitrogen (TN) removal rate was strongly and significantly positively correlated with plant height (*R* = 0.90), root length (*R* = 0.88), total phosphorus removal rate (*R* = 0.85), root biomass (*R* = 0.81), fresh weight per plant (*R* = 0.90), total chlorophyll content (*R* = 0.98), soluble sugar content (*R* = 0.87), ether extract content (*R* = 0.84), SOD activity (*R* = 0.84), and CAT activity (*R* = 0.90) (all *p* < 0.05). In contrast, TN removal was significantly negatively correlated with seedling mortality (*R* = −0.91, *p* < 0.05).

Similarly, the total phosphorus (TP) removal rate showed significant positive correlations with root biomass, SOD, POD, and CAT activities, as well as the TN removal rate (all *p* < 0.05). Conversely, TP removal was significantly negatively correlated with mortality (*R* = −0.94, *p* < 0.05).

Overall, these findings suggest that higher nutrient removal efficiency is closely linked to improved growth, photosynthetic capacity, and antioxidant activity, whereas increased mortality reduces the potential for nutrient uptake.

### 3.8. Comprehensive Evaluation of Barley Seedling Responses to Nitrogen and Phosphorus Concentrations

**Figure 7:**
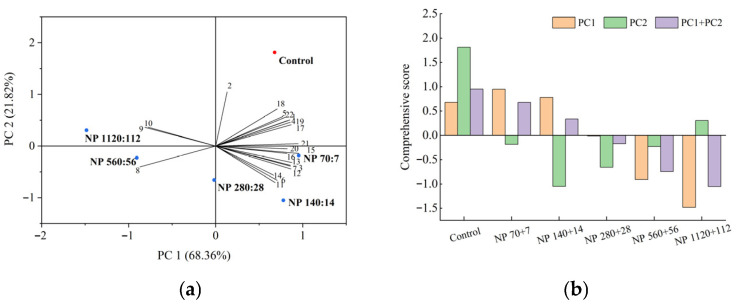
Principal component analysis (PCA) and comprehensive evaluation of barley seedlings under different nitrogen and phosphorus (NP) concentrations. (**a**) PCA biplot showing the distribution of treatments (Control, NP 70 + 7, NP 140 + 14, NP 280 + 28, NP 560 + 56 NP, 1120 + 112 mg·L^−1^) and their associated traits. The first two principal components (PC1 and PC2) explained 68.36% and 21.82% of the total variance, respectively, accounting for a cumulative contribution of 90.18%. (**b**) Comprehensive evaluation scores of barley seedlings based on PC1, PC2, and their combined contribution. Higher scores indicate better overall growth performance, nutritional quality, photosynthetic pigment content, antioxidant activity, and nutrient removal efficiency under specific NP treatments.

Principal component analysis (PCA) was performed to comprehensively evaluate the effects of nitrogen and phosphorus levels on barley seedlings using 22 indicators, including photosynthetic pigments, antioxidant enzyme activities, and nutrient removal efficiencies (Figure 7a). Two principal components with eigenvalues greater than one were extracted, explaining 68.4% and 21.8% of the total variance, respectively, with a cumulative contribution of 90.2%.

The comprehensive scores derived from these two components showed clear treatment differences (Figure 7b). The control group showed the highest score, indicating the most favorable physiological status, followed by NP 7 + 7 and NP 140 + 14, which also performed relatively well. In contrast, the NP 1120 + 112 treatment received the lowest score, reflecting severe stress and reduced overall performance.

These results suggest that moderate nitrogen and phosphorus supplementation (NP 70 + 7 and NP 140 + 14) can enhance multiple physiological traits, whereas excessive nutrient levels (NP 1120 + 112) compromise seedling growth, metabolism, and nutrient uptake.

## 4. Discussion

In this study, we investigated the tolerance of barley seedlings to elevate nitrogen (N) and phosphorus (P) concentrations, identifying thresholds beyond which growth and physiology were adversely affected. By connecting growth performance to physiological responses like antioxidant enzyme activity and changes in photosynthetic pigments, we shed light on how plants adapt when they don’t have enough nutrients. Furthermore, the dual role of barley seedlings as a phytoremediation agent and forage crops was evaluated, emphasizing their potential for sustainable agriculture and ecological restoration.

### 4.1. Early Germination: Delay Rather than Failure

On day 1, the enriched solutions depressed germination relative to the control; however, by day 3, the treatment differences had disappeared. This pattern suggests delayed imbibition and radicle protrusion under high ionic strength rather than a loss of seed viability [30]. Several non-exclusive mechanisms might have been involved in delayed germination, such as osmotic effects, ion-specific effects, or embryo protection. Elevated solute concentrations induce an osmotic effect by reducing the water potential gradient into the seed, slowing water uptake needed to activate metabolism.

Higher nitrogen (ammonium or nitrate) and phosphate can induce ion specific effects by transiently altering the ABA/GA balance that regulates endosperm weakening and radicle emergence. Furthermore, seeds could have arrested development briefly under atypical chemistry, resuming once internal homeostasis was restored. The germination recovery by day 3 indicates that barley seeds delayed but tolerated the NP enrichment levels. This means that both short term and cumulative germination metrics should be used to indicate better establishment potential [30].

### 4.2. Seedling Growth and Mortality: A Narrow Optimum Under N–P Enrichment

Shoot traits (shoot length, shoot fresh mass, total fresh mass) followed a unimodal curve with maxima at NP 70 + 7 (significantly similar at NP 140 + 14 and NP 280 + 28) and showed a reduction at higher concentrations (NP 560 + 56 and 1120 + 112). Compared with standard hydroponic formulations (200–230 mg N·L^−1^; 31 mg P·L^−1^), the treatments, NP 70 + 7 to NP 280 + 28, range from normal to slightly supra optimal supply, whereas NP 560 + 56 and NP 1120 + 112 greatly exceeded typical requirements. Consequently, the higher treatments resulted in growth reduction and exhibited stress responses. In contrast, root length and root mass declined in a monotonic manner as enrichment increased. Seedling mortality increased in parallel with enrichment and was greatest at NP 1120 + 112.

Two mechanisms could explain the change in growth. First, disequilibrium in the carbon–nutrient balance occurs at higher N levels. At low to moderate N–P levels, nutrient addition alleviates the limitations on protein synthesis and chlorophyll formation, enhancing photosynthesis and shoot growth) [28]. As concentrations rise, assimilatory costs (e.g., detoxifying excess ammonium, maintaining ion gradients, storing surplus N–P) divert carbon and energy from growth. The shift toward shoot preference and root suppression mirrors a typical response when nutrients are abundant [30,43]. Under excessive enrichment, ion toxicity and osmotic stress cancel these gains, explaining the downturn in shoot traits [30].

At high NP enrichment, metabolic imbalance (C/N disequilibrium) may contribute to impaired growth and stress symptoms. Downregulation of signaling pathways, such as ATL31 phosphorylation, may explain why barley seedlings fail to efficiently assimilate excess nitrogen [44].

The second mechanism relates to toxicity thresholds and membrane integrity. High N–P levels can disrupt cellular homeostasis, through ammonium or urea derived nitrogen, causing cytosolic acidification [45], or through phosphate overload, which disturbs intracellular phosphate balance and signaling [43]. Such imbalances elevate reactive oxygen species, leading to mortality and reduced growth [46,47]. These results indicate that NP ≤ 280 + 28 supports normal whereas growth ≥ NP 560 + 56 increases seedling loss. The tolerance threshold of barley (NP 280 + 28) is thus defined not only by nutrient imbalance but also by the capacity of its antioxidant system to maintain redox balance.

### 4.3. Nutritional Quality: Improvement at Moderate NP, Decline Under Excess

From a forage quality perspective, crude protein (CP), ether extract (EE), soluble sugars, and dry matter (DM) increased at moderate NP levels and declined at higher concentrations. In contrast, fiber fractions (NDF and ADF) showed the opposite trend, reaching their lowest values at NP 140 + 14. The higher CP content under NP 140 + 14 reflects more efficient nitrogen use for amino acid and protein synthesis, aligning with [48], who found that larch seedlings accumulated more soluble proteins under moderate N and P supply but declined when nutrient levels became excessive.

At moderate N, plants produce more proteins, fats, and sugars, while allocating less to structural materials such as cellulose and lignin [49]. This shift improves digestibility for livestock, as cell contents are more nutritious and easier to digest than fiber [23]. At higher NP levels, however, stress slows growth and redirects carbon toward maintenance and defense, causing fiber content to rise again and reducing forage quality [50]. Overall, the optimal nutritional profile occurred at NP 70 + 7 and NP 140 + 14, corresponding with the most favorable growth performance, and highlighting the dual benefit of these treatments for both plant production and water remediation.

### 4.4. Photosynthetic Pigments: Chlorophyll Is Sensitive, Carotenoids Are Conservative

Chlorophyll a and b increased under low enrichment and then declined at higher levels; carotenoids peaked at NP 70 + 7 and decreased more gradually, remaining above control in most treatments. The decline in chlorophyll content at high NP levels despite nitrogen’s structural role in chlorophyll can be attributed to stress related mechanisms. Similar patterns have been observed, in Larix olgensis and aquatic [51]. Moderate nutrient supply enhanced pigments, but excessive enrichment reduced them, paralleling reduced ALA synthesis and photosynthetic efficiency. Excess nutrients cause ionic stress and reactive oxygen species, damaging chlorophyll and photosystems, particularly PSII, leading to pigment degradation [30].

Carotenoids, in contrast, are more stable and function as photoprotective antioxidants. Their relative stability under stress suggests a role in maintaining photosystem protection even as chlorophyll declines.

### 4.5. Antioxidant System: Enzymes Decline with Enrichment, GSH Peaks at Moderate NP

SOD, POD, and CAT activities declined with increasing N–P levels, with the highest activities in the control. This indicates that excessive enrichment impairs enzymatic ROS scavenging capacity, rather than upregulating it [51]. Disturbed pH homeostasis may alter enzyme conformation or resource allocation from defense to stress tolerance. Similar declines were observed in Vallisneria natans [52] and ryegrass [30]. Some studies noted an initial increase in enzyme activity, followed by a decline at higher nutrient levels [51].

Glutathione (GSH), a key non-enzymatic antioxidant, peaked at NP 140 + 14 before declining. Moderate enrichment likely stimulates anabolic activity, supporting GSH synthesis [53]. At higher NP, oxidative demand exceeds synthesis, and membrane damage disrupts compartmental distribution, lowering GSH pools [30,54]. These data suggest optimal antioxidant health at moderate NP with compromise under extreme enrichment corresponding to growth and pigment responses.

Overall, antioxidant-mediated tolerance in barley seedlings is dual natured. Under moderate eutrophic stress, non-enzymatic antioxidants like GSH are effectively mobilized, maintaining cellular homeostasis and growth. Under severe nutrient overload, enzymatic antioxidants are overwhelmed, leading to ROS accumulation, chlorophyll degradation, and mortality (NP ≥ 560 + 56), limiting phytoremediation efficiency.

### 4.6. Nutrient Removal: Nitrogen Shows an Optimum, Phosphorus Declines with Loading

Barley seedlings removed 29.7−53.4% total N and 47.9−72.9% total P under low-to-moderate enrichment, with efficiency declining under higher nutrient loads. Similar patterns occur in other plant-based systems, such as Sesuvium portulacastrum [55], duckweed [56], and [28]. Even mixed macrophyte systems achieved significant N and P removal [57].

For nitrogen, uptake occurs through active transporters and the NR/NiR/GS−GOGAT pathway coordinated with gene expression [56,58]. At moderate NP, barley rapidly assimilates nitrogen sustaining tissue. At elevated levels, nutrient toxicity, and transporter downregulation reduce nitrogen efficiency. For phosphorus, reduced removal under high loading arises from regulatory feedback limiting phosphate uptake [59,60,61]. Hydroponic conditions lacking mineral sorption sink, exacerbate P accumulation stress, and incrementality [62]. The negative correlation between P removal and mortality supports this interpretation.

Overall, barley seedlings remove phosphorus more effectively than nitrogen across enrichment gradients. Nitrogen removal peaked under low−moderate enrichment (NP 70 + 7 to NP 140 + 14), while phosphorus removal remained high up to NP 280 + 28 before declining. At excessive enrichment (NP 560 + 56 and NP 1120 + 112), both declined, largely due to elevated mortality and suppressed physiological activity [30].

### 4.7. Correlation Patterns and PCA: A Coherent Physiological Signal

Correlation analysis revealed strong relationships between nitrogen removal and plant height, root length, biomass, chlorophyll content, soluble sugars, and antioxidant enzyme activity, and a negative correlation with mortality. Phosphorus removal showed a similar pattern. These findings highlight that nutrient removal efficiency depends on physiological vigor [57]. PCA of 22 traits explained ~90% of variance, with moderate NP treatments and controls showing high performance, while NP 1120 + 112 was associated with stress. This confirms that low to moderate nutrient enrichment yields the most favorable plant growth and remediation outcomes.

### 4.8. Implications for Phytoremediation and Forage Coproduction

Barley seedlings can be effectively used in nutrient enriched waters, if nitrogen and phosphorus concentrations do not exceed NP 280 + 28. Within this range, plants maintained good growth, high nutritional quality, and efficient nutrient removal.

At higher concentrations (NP 560 + 56 and NP 1120 + 112), multiple stress reduced pigments, lower antioxidant activity, increased mortality, and reduced nutrient removal were evident. Thus, barley alone is unlikely to sustain remediation under extreme enrichment. The forage produced at optimal NP (70 + 7 to 140 + 14) had favorable nutritional quality, supporting dual benefits: nutrient removal from eutrophic water and production of high-quality feed. Future studies should evaluate barley’s performance in natural eutrophic systems to validate these findings under variable environmental conditions.

## 5. Conclusions

This study demonstrated that barley seedlings tolerate and perform well under low to moderate N–P enrichment (≤NP 280 + 28). Within this range, they exhibited vigorous growth, with favorable forage quality (higher crude protein and soluble sugars, lower fiber fractions), stable pigment and antioxidant status, and effective nutrient removal. At higher concentrations (NP 560 + 56 and NP 1120 + 112), stress responses became evident, including reduced chlorophyll and antioxidant activity, increased mortality, and markedly lower N and P removal efficiency. This study highlights the potential of hydroponic barley as a dual-purpose strategy for water remediation and forage production, but several constraints remain. Conducted in a controlled nutrient solution, it did not capture the organic matter, metals, and pH fluctuations of natural waters. Only one cultivar (Ganpi No. 4) was tested, and the seven-day trial did not assess longer-term dynamics or repeated harvests. Future studies should therefore extend the experimental duration, compare diverse cultivars, and test performance in real eutrophic waters to better define the practical scope of this approach.

## Figures and Tables

**Table 1:** Effects of different nitrogen and phosphorus levels on germination rate of barley seeds.

Treatment	Germination Rate on the First Day (%)	Germination Rate on the Third Day (%)
Control	51.17 ± 0.73 ^a^	70.00 ± 0.58 ^a^
NP 70:7	38.83 ± 2.68 ^b^	68.00 ± 2.65 ^a^
NP 140:14	38.00 ± 1.53 ^b^	67.33 ± 3.18 ^a^
NP 280:28	29.33 ± 1.45 ^c^	67.67 ± 2.03 ^a^
NP 560:56	28.67 ± 2.62 ^c^	67.67 ± 2.40 ^a^
NP 1120:112	18.67 ± 3.28 ^d^	68.33 ± 0.88 ^a^

Treatments: Control (distilled water); NP 70:7 = 70 mg·L^−1^ N + 7 mg·L^−1^ P; NP 140:14 = 140 mg·L^−1^ N + 14 mg L^−1^·P; NP 280:28 = 280 mg·L^−1^ N + 28 mg·L^−1^ P; NP 560:56 = 560 mg·L^−1^ N + 56 mg·L^−1^ P; NP 1120:112 = 1120 mg·L^−1^ N + 112 mg·L^−1^. *p* Values represent mean ± SE (n = 3). Different letters indicate significant differences among treatments according to Tukey’s HSD test (*p* < 0.05).

## Data Availability

The data presented in this study are available on request from the corresponding author.

## Abbreviations

The following abbreviations are used in this manuscript:

ADF	Acid detergent fiber
CAT	Catalase
CP	Crude protein
DM	Dry matter
EE	Ether extract
FW	Fresh weight
GSH	Glutathione
NDF	Neutral detergent fiber
POD	Peroxidase
SOD	Superoxide dismutase
TN	Total nitrogen
TP	Total phosphorus

## References

[1] Li H., Chen Y. (2020). Assessing potential land suitable for surface irrigation using groundwater data and multi-criteria evaluation in Xinjiang Inland River Basin. Comput. Electron. Agric..

[2] Geng Q., Zhao Y., Sun S., He X., Wang D., Wu D., Tian Z. (2023). Spatio-temporal changes and its driving forces of irrigation water requirements for cotton in Xinjiang, China. Agric. Water Manag..

[3] Chu J.Q., Jiang Z.H. (2024). Spatiotemporal evolution and influencing factors of green efficiency of agricultural water resources in Xinjiang. Arid Land Geogr.

[4] Li L., Wang S., Chen Y., Zhang H., Zhang J., Xu Y., Wei J. (2023). Climate change in the eastern Xinjiang of China and Its connection to northwestern warm humidification. Atmosphere.

[5] Xin L. (2022). Chemical fertilizer rate, use efficiency and reduction of cereal crops in China, 1998–2018. J. Geogr. Sci..

[6] Ma H., Pu S., Li P., Niu X., Wu X., Yang Z., Zhu J., Yang T., Hou Z., Ma X. (2021). Towards to understanding the preliminary loss and absorption of nitrogen and phosphorus under different treatments in cotton drip-irrigation in northwest Xinjiang. PLoS ONE.

[7] Kong M., Guan Q., Feng L., Zheng C., Tang J. (2025). Changes in land-leaching nitrogen and its linkages to lake algal blooms in China. Environ. Res. Ecol..

[8] Ni Z., Wang S. (2015). Economic development influences on sediment-bound nitrogen and phosphorus accumulation of lakes in China. Environ. Sci. Pollut. Res..

[9] Kapsalis V.C., Kalavrouziotis I.K. (2021). Eutrophication—A worldwide water quality issue. Chemical Lake Restoration: Technologies, Innovations and Economic Perspectives.

[10] Liu X., Sathishkumar K., Zhang H., Saxena K.K., Zhang F., Naraginti S., Rajendiran R., Rajasekar A., Guo X. (2024). Frontiers in environmental cleanup: Recent advances in remediation of emerging pollutants from soil and water. J. Hazard. Mater. Adv..

[11] El-Sheekh M., Abdel-Daim M.M., Okba M., Gharib S., Soliman A., El-Kassas H. (2021). Green technology for bioremediation of the eutrophication phenomenon in aquatic ecosystems: A review. Afr. J. Aquat. Sci..

[12] Gao G., Xiong H., Chen J., Chen K., Chen P., Yu C., Zhu A. (2018). Hydroponic method for ramie and removal of nitrogen and phosphorus from livestock wastewater. Int. J. Phytoremediation.

[13] Ting W.H.T., Tan I.A.W., Salleh S.F., Wahab N.A. (2018). Application of water hyacinth (*Eichhornia crassipes*) for phytoremediation of ammoniacal nitrogen: A review. J. Water Process Eng..

[14] Wang X., Jain A., Chen B., Wang Y., Jin Q., Yugandhar P., Xu Y., Sun S., Hu F. (2022). Differential efficacy of water lily cultivars in phytoremediation of eutrophic water contaminated with phosphorus and nitrogen. Plant Physiol. Biochem..

[15] Nahar K., Sunny S.A. (2024). Co-benefits of *Eichhornia crassipes* (water hyacinth) as sustainable biomass for biofuel production and aquatic ecosystem phytoremediation. Fuels.

[16] Xu X., Zhou Y., Han R., Song K., Zhou X., Wang G., Wang Q. (2019). Eutrophication triggers the shift of nutrient absorption pathway of submerged macrophytes: Implications for the phytoremediation of eutrophic waters. J. Environ. Manag..

[17] Wu K., Chen L., Wang Q., Li Y., Zheng Y., Ma Q., Li H., Zhang Y., Li F. (2025). Seasonal Dynamics of Nitrogen and Phosphorus in Wetland Plants: Implications for Efficient Eutrophication Control. Sustainability.

[18] Phang L.Y., Mingyuan L., Mohammadi M., Tee C.S., Yuswan M.H., Cheng W.H., Lai K.S. (2024). Phytoremediation as a viable ecological and socioeconomic management strategy. Environ. Sci. Pollut. Res..

[19] Ghaly A., Kamal M., Mahmoud N. (2004). Phytoremediation of aquaculture wastewater for water recycling and production of fish feed. Environ. Int..

[20] Dabney M.S., Delgado A.J., Reeves W.D. (2001). Using winter cover crops to improve soil and water quality. Commun. Soil Sci. Plant Anal..

[21] Rabêlo F.H.S., Vangronsveld J., Baker A.J., van Der Ent A., Alleoni L.R.F. (2021). Are grasses really useful for the phytoremediation of potentially toxic trace elements? A review. Front. Plant Sci..

[22] dos Santos N., Clyde-Smith D., Qi Y., Gao F., Busquets R., Campos L.C. (2023). A study of microfiber phytoremediation in vertical hydroponics. Sustainability.

[23] Ma Y., Khan M.Z., Liu Y., Xiao J., Chen X., Ji S., Cao Z., Li S. (2021). Analysis of nutrient composition, rumen degradation characteristics, and feeding value of Chinese rye grass, barley grass, and naked oat straw. Animals.

[24] Gebremedhin W.K. (2015). Nutritional benefit and economic value of feeding hydroponically grown maize and barley fodder for Konkan Kanyal goats. IOSR J. Agric. Vet. Sci.

[25] Fazaeli H., Golmohammadi H.A., Tabatabayee S.N., Asghari-Tabrizi M. (2012). Productivity and nutritive value of barley green fodder yield in hydroponic system. World Appl. Sci. J..

[26] Al-Ajmi A., Salih A., Kadhim I., Othman Y. (2009). Yield and water use efficiency of barley fodder produced under hydroponic system in GCC countries using tertiary treated sewage effluents. J. Phytol..

[27] Adrover M., Moyà G., Vadell J. (2013). Use of hydroponics culture to assess nutrient supply by treated wastewater. J. Environ. Manag..

[28] Ding Z., Golan-Goldhirsh A., Rafiq M.K., Li T., Zhao F., Yang X. (2012). Purification of eutrophic water by ryegrass. Water Sci. Technol..

[29] Li M., Sheng G.P., Wu Y.J., Yu Z.L., Bañuelos G.S., Yu H.Q. (2014). Enhancement of nitrogen and phosphorus removal from eutrophic water by economic plant annual ryegrass (*Lolium multiflorum*) with ion implantation. Environ. Sci. Pollut. Res..

[30] Xu H.S., Zhu L., Mei Y. (2021). Effects of high levels of nitrogen and phosphorus on perennial ryegrass (*Lolium perenne* L.) and its potential in bioremediation of highly eutrophic water. Environ. Sci. Pollut. Res..

[31] (2002). Environmental Quality Standards for Surface Water.

[32] Lichtenthaler H.K., Wellburn A.R. (1983). Determinations of total carotenoids and chlorophylls a and b of leaf extracts in different solvents. Biochem. Soc. Trans..

[33] Giannopolitis C.N., Ries S.K. (1977). Superoxide dismutases: I. Occurrence in higher plants. Plant Physiol..

[34] Chance B., Maehly A.C. (1955). Assay of catalases and peroxidases. Methods Enzymol..

[35] Aebi H. (1984). Catalase in vitro. Methods Enzymol..

[36] Bremner J.M., Mulvaney C.S., Page A.L., Miller R.H., Keeney D.R. (1982). Nitrogen—Total. Methods of Soil Analysis, Part 2: Chemical and Microbiological Properties.

[37] Murphy J., Riley J.P. (1962). A modified single solution method for the determination of phosphate in natural waters. Anal. Chim. Acta.

[38] (2016). Official Methods of Analysis of AOAC International.

[39] (2014). Determination of Moisture in Feedstuffs.

[40] Van Soest P.J., Robertson J.B., Lewis B.A. (1991). Methods for Dietary, Neutral Detergent Fiber and Nonstarch Polysaccharides in Relation to Animal Nutrition. J. Dairy Sci..

[41] (2018). Determination of Crude Protein in Feeds–Kjeldahl Method.

[42] (2025). Determination of Crude Fat in Feeds.

[43] Chen G., Li Y., Jin C., Wang J., Wang L., Wu J. (2021). Physiological and morphological responses of hydroponically grown pear rootstock under phosphorus treatment. Front. Plant Sci..

[44] Osuna D., Prieto P., Aguilar M. (2015). Control of seed germination and plant development by carbon and nitrogen availability. Front. Plant Sci..

[45] Du W., Zhang Y., Si J., Zhang Y., Fan S., Xia H., Kong L. (2021). Nitrate alleviates ammonium toxicity in wheat (*Triticum aestivum* L.) by regulating tricarboxylic acid cycle and reducing rhizospheric acidification and oxidative damage. Plant Signal. Behav..

[46] Takagi D., Miyagi A., Tazoe Y., Suganami M., Kawai-Yamada M., Ueda A., Suzuki Y., Noguchi K., Hirotsu N., Makino A. (2020). Phosphorus toxicity disrupts Rubisco activation and reactive oxygen species defence systems by phytic acid accumulation in leaves. Plant Cell Environ..

[47] Lambers H. (2022). Phosphorus acquisition and utilization in plants. Annu. Rev. Plant Biol..

[48] Chu D.M., Li T.K., Yin Y.L. (2006). Effects of dietary crude protein levels on growth performance, nitrogen utilization and carcass characteristics of growing-finishing pigs. Asian-Australas. J. Anim. Sci..

[49] Tahir M., Wei X., Liu H., Li J., Zhou J., Kang B., Jiang D., Yan Y. (2023). Mixed legume–grass seeding and nitrogen fertilizer input enhance forage yield and nutritional quality by improving the soil enzyme activities in Sichuan, China. Front. Plant Sci..

[50] Eltelib H.A., Ali E.E. (2006). Effect of time of nitrogen application on growth, yield and quality of four forage sorghum cultivars. Agric. J..

[51] Xiao H., Peng S., Liu X., Jia J., Wang H. (2021). Phytoremediation of nutrients and organic carbon from contaminated water by aquatic macrophytes and the physiological response. Environ. Technol. Innov..

[52] Wang C., Zhang S.H., Wang P.F., Hou J., Li W., Zhang W.J. (2008). Metabolic adaptations to ammonia-induced oxidative stress in leaves of the submerged macrophyte *Vallisneria natans* (Lour.) Hara. Aquat Toxicol.

[53] Esposito S. (2016). Nitrogen assimilation, abiotic stress and glucose 6-phosphate dehydrogenase: The full circle of reductants. Plants.

[54] Zhang Y., Luo P., Zhao S., Kang S., Wang P., Zhou M., Lyu J. (2020). Control and remediation methods for eutrophic lakes in the past 30 years. Water Sci. Technol..

[55] Zhang C., Wang D., He W., Liu H., Chen J., Wei X., Mu J. (2022). Sesuvium portulacastrum-mediated removal of nitrogen and phosphorus affected by sulfadiazine in aquaculture wastewater. Antibiotics.

[56] Zhu Q., Li Y., Hassan M.A., Fang W., Wang S. (2024). Comparative physiological and transcriptomic characterization of rice (*Oryza sativa L.*) root seedlings under phosphorus deficiency. Plant Stress.

[57] Wang X., Wang Y., Yao W., Shangguan L., Zhang X., Jin Q., Cong X., Qian P., Xu Y. (2023). Improving the efficacy of different life-form macrophytes in phytoremediation of artificial eutrophic water by combined planting. Environ. Sci. Pollut. Res..

[58] Fan X., Lu C., Khan Z., Li Z., Duan S., Shen H., Fu Y. (2025). Mixed ammonium-nitrate nutrition regulates enzymes, gene expression, and metabolic pathways to improve nitrogen uptake, partitioning, and utilization efficiency in rice. Plants.

[59] Prathap V., Kumar A., Maheshwari C., Tyagi A. (2022). Phosphorus homeostasis: Acquisition, sensing, and long-distance signaling in plants. Mol. Biol. Rep..

[60] Bayle V., Arrighi J.F., Creff A., Nespoulous C., Vialaret J., Rossignol M., Gonzalez E., Paz-Ares J., Nussaume L. (2011). Arabidopsis thaliana high-affinity phosphate transporters exhibit multiple levels of posttranslational regulation. Plant Cell.

[61] Nussaume L., Kanno S., Javot H., Marin E., Pochon N., Ayadi A., Nakanishi T.M., Thibaud M.C. (2011). Phosphate import in plants: Focus on the PHT1 transporters. Front. Plant Sci..

[62] Manzoor A. (2024). Dynamics of Phosphorus Availability and Uptake in Hydroponics and Soils. Ph.D. Thesis.

